# Maternal postpartum feeding anxiety was associated with infant feeding practices: results from the mother-infant cohort study of China

**DOI:** 10.1186/s12884-020-03483-w

**Published:** 2020-12-14

**Authors:** Jing Sun, Yimin Zhu, Yongjin Li, Niuniu Li, Tan Liu, Xiao Su, Zhiyong Dai, Yanchun Zhang, Lina Pan, Wei Jiang, Wenli Zhu

**Affiliations:** 1 Department of Nutrition and Food Hygiene, Peking University School of Public Health; National Health Commission Key Laboratory of Reproductive Health, Beijing, 100191 China; 2 Shijingshan District Center for Diseases Prevention and Control, Beijing, 100043 China; 3 Shunyi District Center for Diseases Prevention and Control, Beijing, 101300 China; 4 Peking-Ausnutria Maternal and Infant Nutrition Research Center, Beijing, 100191 China

**Keywords:** Maternal, Postpartum feeding anxiety, Infant feeding practices, Breastfeeding

## Abstract

**Background:**

Maternal feeding anxiety (FA) was prevalent during puerperium and might affect infant feeding practices. This study was aimed to investigate the FA status in Chinese postpartum women and its relationship with infant feeding practices (FPs).

**Methods:**

Participants were from the Mother-Infant Cohort Study of China, in which the dietary and feeding practices, physical and psychiatric health for both mothers and infants were followed up from childbirth to next 2 years. In this study the maternal feeding anxiety (FA) status at 0–3 months postpartum was assessed by Li’s Self-rating Feeding Anxiety Scale (SFAS). Infant feeding practices (FPs) at 0–3 months, including breastfeeding-related behaviors, responsive feeding and infant food refusal were investigated by self-designed questionnaire.

**Results:**

In total 456 mothers the average feeding anxiety scores (FAS) was 41.02 ± 8.02 (mean ± SD), and maternal FA prevalence were 61.4% (FAS>38) with severe FA being 8.6% (FAS>52) at 0–3 months postpartum. The FAS was related with infant FPs, and lower maternal FAS was significantly related with infant colostrum feeding (40.86 ± 8.02 vs 44.74 ± 11.33, *P* < 0.05), but higher FAS was related with bottle feeding (41.95 ± 8.28 vs 39.69 ± 7.92, *P* < 0.05). The mothers with severe feeding anxiety (FAS > 53) were more likely to feed infants with bottle (ORs, 95%CI: 2.41, 1.11 ~ 5.19). There were not significant association between FAS and exclusive breastfeeding and responsive feeding practices (*P* > 0.05). The higher FAS was associated with infant food refusal behaviors, the maternal scores whose infant “never”, “rarely”, “sometimes” and “often” spat out food when feeding were 39.86 ± 8.02, 41.47 ± 8.18, 41.36 ± 7.44 and 42.14 ± 12.03 increasingly (*P* > 0.05), and the FA prevalence was significantly different among groups (*P* < 0.05). The infants whose mother was identified as feeding anxiety were more likely to refuse opening the mouth when feeding (*P* < 0.05). Multivariate analysis indicated maternal FAS was positively related to infant bottle feeding (βi = 2.487, *P* < 0.05) and outdoor sunshine exposure practice (βi = 1.787, *P* < 0.05), and negatively related to household income level (βi = − 0.118, *P* < 0.05).

**Conclusions:**

Maternal postpartum feeding anxiety was associated with some infant feeding practices, including bottle feeding and infant food refusal behaviors.

**Supplementary Information:**

The online version contains supplementary material available at 10.1186/s12884-020-03483-w.

## Background

While the physical health of women and children is emphasized, the mental aspects of their health are often ignored, especially in low- and middle-income countries (LMICs). Literatures showed that perinatal anxiety and depression are more prevalent in women from LMICs, being 15.6% during the prenatal period and 19.8% during the postnatal period, as compared to high-income countries, ranging from 6.5 to 12.9% during the perinatal period [1]. Globally, in women of childbearing age, postpartum psychiatric symptoms including anxiety and depression account for the largest proportion of the burden associated with mental or neurological disorders. In addition to the economic and human costs of maternal depression, children of mothers who are depressed are at risk for poor health, developmental, and behavioral problems, thereby contributing to intergenerational disadvantage that accumulates throughout the life span.

The aetiology of perinatal mental health problems is multifactorial, with studies consistently reporting the importance of psychosocial variables such as life stress and lack of social support. In China postpartum women participate in a traditional ancient practice called “zuoyuezi” or “doing the month”, including special diet, maintaining warmth, restricting physical and outdoor activity. During the puerperium, not only the limited socialization, but also the contradiction of adherence to traditional beliefs supported by family elders and modern scientific guideline, may increase the postpartum psychiatric symptoms risk in Chinese women. A longitudinal cohort study on postpartum depression and anxiety among 571 Chinese immigrant and nonimmigrant women with live births in 2011 to 2014 showed that Chinese immigrant women are a high-risk group for postpartum depressive and anxiety symptomatology, possible depressive symptomology was most prevalent and occurred in a quarter of women at 4 weeks postpartum (24.4%; 95% CI, 21.1–28.2%) and decreased to 17.9% (95% CI, 14.6–21.8%) at 52 weeks postpartum [2].

Poor maternal mental health is not only detrimental to the mother’s physical and psychological health, but also has adverse impact on her infant’s feeding practices (FPs), and ultimate growth and development. Feeding anxiety (FA) refers to an unpleasant emotional state correlated with infant FPs and outcome, which is a dimension of postnatal anxiety. FA also diminishes maternal breastfeeding confidence and self-efficacy, and can lead to a reduced duration of breastfeeding or a decline in exclusive breastfeeding [3, 4]. A systematic review provided evidence for the effect of negative postpartum mood on breastfeeding, showed that women with symptoms of postpartum anxiety are less likely to breastfeed exclusively and initiate breastfeeding, and more likely to terminate breastfeeding earlier and supplement with formula in the hospital [5]. Heterogeneous outcomes and methodological limitations somewhat limit the comparability of findings.

Overall, The first 3 months after delivery is a critical window period for the infants, during which the maternal emotional status directly affect infant’s feeding behaviors, physical and mental development, and furtherly the adulthood health [4, 6]. It is therefore highly importance to identify and improve maternal FA to avoid its negative effects on infant FPs and development. But to now FA was rarely concerned in China. The aim of this study was to investigate the FA status in Chinese puerperal women and its relationship with infant FPs, to provide the targets for further maternal mental health program.

## Methods

### Participants

This study used data collected as part of the Mother-Infant Cohort Study (MICS) of China. The MICS was conducted from June 2015 to December 2018 in seven cities (Beijing, Taiyuan of Shanxi, Jinan of Shandong, Changsha of Hunan, Shiyan of Hubei, Chongqing, and Chengdu of Sichuan) from northern, eastern, central and southwestern districts around China. At baseline mother-infant pairs at 0–3 months postpartum were voluntarily recruited from local child care clinics, and then followed up to 2 years. The mothers were healthy and the neonates were born without severe deformities. Participants with incomplete information or logical error were removed, and 490 mother-infant pairs were included in the study and 456 questionnaires (93.1%) were available for the final analysis. The data of the study was investigated at 0–3 months postpartum.

The study was approved by the Peking University Institutional Review Board (Beijing, China) and informed written consent was obtained from all mother participants.

### Data collection

#### Maternal feeding anxiety (FA) assessment

Maternal FA was assessed using Li’s Self-rating Feeding Anxiety Scale (SFAS) [7] at 0–3 months postpartum. The SFAS (2013) scale consists of four dimensions (worrying, self-confidence, annoying and stress) and 23 items. The mothers were asked some infant feeding-related questions, like “I was annoyed by infant’s crying.” “I was competent to be a mother.” “I worried about child appetite.” “I was stressed about infant food safety.” and so on. All items are rated on a 4-point Likert scale to produce a summative score ranging from 23 to 92, with higher scores indicating increased anxiety symptom. The SFAS has been used broadly in studies of Chinese postpartum women’s feeding anxiety, while a recommended cutoff of 38 [8]. The reliability and validity of SFAS were tested and Cronbach’s alpha coefficient was 0.829 and the internal consistency coefficient was 0.730. In this study we defined FAS > 38 being mild feeding anxiety and FAS > 53 being severe anxiety (Supplementary file 1).

#### Infant feeding practices (FPs) investigation

Infant feeding practices (FPs) were defined as a series of behaviors and related practices exhibited by infants and mothers, that affected infant’s food and nutrients intake directly or indirectly, including feeding pattern, bottle feeding, responsive feeding, mother-child interactions, food refusal, etc.

The FPs investigated in the study included: 1) Breastfeeding related practices, including touching breast in mother’s arms within 1 h after delivery; colostrum feeding (breastfeeding within 7 days after delivery); feeding other food or liquid (excluding milk) before breastfeeding within 3 days after delivery; exclusive breastfeeding (with allowance for oral rehydration salts, vitamins, minerals, medications, and infusion); feeding water, beverages, or juice; feeding infant formula; bottle feeding. 2) Responsive feeding practices, including perception of infant hungry and satiety signs, on-demand feeding, fixed-time feeding. 3) Infant’s food refusal behaviors, including refusal to open the mouth, turning head aside, crying when feeding. 4) Outdoor sunshine exposure of infant. Mothers answered the self-designed questionnaire at 0–3 months postpartum and the options included different levels like “never”, “rarely”, “sometimes”, “often”, and “yes” or “no” (Supplementary file 2).

#### Covariates

Demographic characteristics were measured at the 0–3 months postpartum interview, including maternal age, child-bearing number, marital status, education level, household income (monthly income less than 3000 yuan per capita was defined as “low” level, 3000–6000 yuan being “middle”, more than 6000 yuan being “high”). Gestational status and delivery mode were also investigated by questionnaire (Supplementary file 3).

Local physicians and nurses distributed the self-reported questionnaire to the women at 0–3 months postpartum, provided explanations face to face or by phone, and guided them to fill out the questionnaire.

#### Statistical analysis

The EpiData3.1 software was used for data input and the SPSS 20.0 software was used for statistical analysis. Prevalence and score (mean ± SD) of maternal feeding anxiety were calculated. Differences of FAS and FA prevalence were compared among different demographic characteristics and gestational and delivery status using t-test, one-way analysis of variance and chi-squared test. To analyze the relationship between maternal FA and infant FPs, multiple linear regression was used to calculate odds ratios (ORs) with 95% confidence intervals (95% CIs). Two-tailed *P*-values < 0.05 were considered statistically significant.

## Results

### Characteristics of participants

The maternal age in the study was from 21 to 42 years. A total of 321 (70.4%) women were first-time mother and 20 (4.4%) women delivered preterm birth (< 37 weeks). In addition, 271 (59.4%) women had vaginal delivery and others (40.6%) delivered by cesarean section. Moreover, 102 (22.4%) infants were breastfed exclusively, as shown in Table 1.

**Table 1:** Characteristics of participants (*n* = 456)

Characteristics	N	Percentage (%)	Characteristics	N	Percentage (%)
Maternal age (years)			Gestational age (weeks)		
21–27	110	24.1	≥37	436	95.6
28–34	277	60.7	< 37	20	4.4
≥ 35	69	15.1			
Educational degree			Delivery mode		
≤ High school	170	37.3	Vaginal	271	59.4
Bachelor	214	46.9	Caesarean	185	40.6
Postgraduate or above	72	15.8			
Household income level			Exclusive breastfeeding		
Low	93	20.4	Yes	102	22.4
Middle	202	44.3	No	354	77.6
High	161	35.3	Feeding anxiety symptom		
Child-bearing number			Negative	176	38.6
1	321	70.4	Mild	241	52.9
≥ 2	135	29.6	Severe	39	8.6

The maternal feeding anxiety was prevalent, and 61.4% (280/456) had anxiety symptom. Among those the prevalence of mild and severe FA symptom was 52.9% (241/456) and 8.6% (39/456), respectively.

The feeding anxiety score (FAS) among 456 postpartum women was 41.02 ± 8.02 (mean ± SD). Differences in FAS and FA prevalence among groups categorized by sociodemographic characteristics were analyzed by one-way ANOVA, as shown in Table 2. In Table 2, the chi square test indicated the delivery mode was associated with postpartum feeding anxiety significantly (*P* < 0.05), but without significance if combining the mild and severe FA as one group (OR = 1.33, 95%CI: 0.90–1.96). Furtherly comparing the mild and severe FA groups with negative group separately, the results showed the maternal mild FA prevalence with caesarean delivery was much higher than those with vaginal delivery (60.0% vs 48.0%, OR = 1.49, 95%CI: 1.00–2.22, *P* < 0.05), but the prevalence of severe FA was not significantly different (5.4% vs 10.7%, OR = 0.60, 95%CI: 0.28–1.32, *P* > 0.05). So the study showed the CS delivery related with postpartum mild FA risk, but couldn’t conclude the CS affect positively severe FA. A higher FAS was found in women with younger age, lower household income, first-time mother and preterm birth, but without statistical significance (*P* > 0.05*)*. FA prevalence was associated with delivery mode (*P* < 0.05*)*.

**Table 2:** Difference in FAS and FA prevalence in relation to different characteristics

Characteristics	N	FAS	*P* value	Feeding anxiety n(%)			*P* value	ORs^a^ (95% CI)
		(mean ± SD)		Negative(≤38)	Mild(39 ~ 52)	Severe(≥53)		
Maternal age (years)								
21–27	110	41.71 ± 8.27	0.487	40 (36. 4%)	60 (54.5%)	10 (9.1%)	0.830	1
28–34	277	40.94 ± 8.12		105 (37.9%)	148 (53.4%)	24 (8.7%)		0.94 (0.59 ~ 1.48)
≥ 35	69	40.23 ± 8.45		31 (44.9%)	33 (47.8%)	5 (7.2%)		0.70 (0.38 ~ 1.29)
Education degree								
≤ High school	170	40.97 ± 7.94	0.951	63 (37.1%)	92 (54.1%)	15 (8.8%)	0.744	1
Bachelor	214	41.13 ± 8.31		81 (37.9%)	116 (54.2%)	17 (7.9%)		0.97 (0.64 ~ 1.47)
Postgraduate or above	72	40.79 ± 8.59		32 (44.4%)	33 (45.8%)	7 (9.7%)		0.74 (0.42 ~ 1.29)
Household income level								
Low	93	42.59 ± 9.48	0.111	33 (35.5%)	50 (53.8%)	10 (10.8%)	0.886	1
Middle	202	40.73 ± 8.12		78 (38.6%)	107 (53.0%)	17 (8.4%)		0.87 (0.52 ~ 1.46)
High	161	40.47 ± 7.40		65 (40.4%)	84 (52.2%)	12 (7.5%)		0.81 (0.48 ~ 1.38)
Parity								
1	321	41.13 ± 8.40	0.659	126 (39.3%)	163 (50.8%)	32 (10.0%)	0.170	1
≥2	135	40.76 ± 7.74		50 (37.0%)	78 (57.8%)	7 (5.2%)		1.10 (0.73 ~ 1.63)
Gestational age (weeks)								
≥ 38	436	40.99 ± 8.19	0.767	168 (38.5%)	231 (53.0%)	37 (8.5%)	0.888	1
< 37	20	41.55 ± 8.59		8 (40.0%)	10 (50.0%)	2 (10.0%)		0.94 (0.38 ~ 2.35)
Delivery mode								
Vaginal	271	41.06 ± 8.42	0.887	112 (40.3%)	130 (48.0%)	29 (10.7%)	0.019*	1
Caesarean	185	40.95 ± 7.88		64 (34.6%)	111 (60.0%)	10 (5.4%)		1.33 (0.90 ~ 1.96)

*FAS* Feeding anxiety score

^a^ ORs value was calculated as combination of mild and severe anxiety (FAS > 38) compared with negative (FA ≤ 38) group

* *P* < 0.05

### Relations of maternal feeding anxiety with infant breastfeeding-related practices

The lower maternal FAS was significantly related with infant colostrum feeding (40.86 ± 8.02 vs 44.74 ± 11.33, *P* < 0.05), but higher FAS was related with infant bottle feeding (41.95 ± 8.28 vs 39.69 ± 7.92, *P* < 0.05) and outdoor sunshine exposure (41.93 ± 8.18 vs. 40.27 ± 8.16, *P* < 0.05), as shown in Table 3. The mothers with severe feeding anxiety (FAS > 53) were more likely to feed infants with bottle (ORs, 95%CI: 2.41, 1.11 ~ 5.19), compared with combination of mild and negative FA. There were not significant association between FA and exclusive breastfeeding (*P* > 0.05).

**Table 3:** Difference in maternal FAS and FA prevalence in relation to breastfeeding-related practices

Breastfeeding-related practices	N	FAS	*P* value	Feeding anxiety n(%)			*P* value	ORs (95% CI) ^**a**^
		(mean ± SD)		Negative(≤38)	Mild(39 ~ 52)	Severe(≥53)		
Touching breast in mother’s arms within 1 h after delivery								
No	197	41.00 ± 8.22	0.968	78 (39.6%)	102 (51.8%)	17 (8.6%)	0.920	1
Yes	259	41.03 ± 8.20		98 (37.8%)	161 (62.2%)	22 (8.5%)		1.08 (0.74 ~ 1.58)
Colostrum feeding								
No	19	44.74 ± 11.33	0.043*	6 (31.6%)	10 (52.6%)	3 (15.8%)	0.376	1
Yes	437	40.86 ± 8.02		170 (38.9%)	231 (52.9%)	36 (8.2%)		0.73 (0.27 ~ 1.94)
Feeding other non-milk food or liquid before breastfeeding after delivery								
No	272	41.19 ± 7.89	0.583	101 (37.1%)	148 (54.4%)	23 (8.5%)	0.707	1
Yes	184	40.76 ± 8.66		75 (40.8%)	93 (50.5%)	16 (8.7%)		0.86 (0.59 ~ 1.26)
Exclusive breastfeeding								
No	354	40.99 ± 8.14	0.911	132 (37.3%)	194 (54.8%)	28 (7.9%)	0.272	1
Yes	102	41.10 ± 8.45		44 (43.1%)	47 (53.9%)	11 (10.8%)		0.78 (0.50 ~ 1.23)
Feeding water, beverages or juice presently								
No	201	40.40 ± 7.73	0.156	78 (38.8%)	109 (54.2%)	14 (7.0%)	0.551	1
Yes	255	41.50 ± 8.54		98 (38.4%)	132 (51.8%)	25 (9.8%)		1.02 (0.70 ~ 1.49)
Feeding infant formula								
No	261	40.74 ± 8.11	0.403	101 (38.7%)	142 (54.4%)	18 (6.9%)	0.327	1
Yes	195	41.39 ± 8.33		75 (38.5%)	99 (50.8%)	21 (10.8%)		1.01 (0.69 ~ 1.48)
Bottle feeding								
No	188	39.69 ± 7.92	0.004*	79 (42.0%)	100 (53.2%)	9 (4.8%)	0.043^*^	1
Yes	268	41.95 ± 8.28		97 (36.2%)	141 (52.6%)	30 (11.2%)		1.28 (0.87 ~ 1.87)
Outdoor sunshine exposure								
No	251	40.27 ± 8.16	0.032*	106 (42.2%)	125 (49.8%)	20 (8.0%)	0.210	1
Yes	205	41.93 ± 8.18		70 (34.1%)	116 (56.6%)	19 (9.3%)		1.41 (0.96 ~ 2.07)

*FAS* Feeding anxiety score

^a^ ORs value was calculated as combination of mild and severe anxiety (FAS > 38) compared with negative (FA ≤ 38) group

* *P* < 0.05

### Relations of maternal feeding anxiety with responsive feeding practices

Differences in FAS between groups categorized by responsive feeding practices were analyzed by one-way ANOVA and were shown in Table 4. However, the study found no significant relationship between responsive feeding practices and maternal FAS and FA prevalence (*P* > 0.05).

**Table 4:** Difference in maternal FAS and FA prevalence in relation to responsive feeding practices

Responsive feeding	N	FAS	*P* value	Feeding anxiety n(%)			*P* value	ORs(95% CI)^a^
		(mean ± SD)		Negative(≤38)	Mild(39 ~ 52)	Severe(≥53)		
Feeding as more as possible								
Never	85	40.58 ± 8.02	0.868	33 (38.8%)	45 (52.9%)	7 (8.2%)	0.996	1
Rarely	74	40.80 ± 8.83		28 (37.8%)	40 (54.1%)	6 (8.1%)		1.04 (0.55 ~ 1.98)
Sometimes	111	40.82 ± 8.10		44 (39.6%)	59 (53.2%)	67 (60.4%)		0.97 (0.54 ~ 1.72)
Often	132	41.68 ± 8.03		71 (38.2%)	97 (52.2%)	82 (62.1%)		1.03 (0.61 ~ 1.74)
Perception of infant hungry and satiety signs								
Never	198	41.13 ± 8.41	0.171	74 (37.4%)	107 (54.0%)	17 (8.6%)	0.934	1
Rarely	224	41.19 ± 8.20		87 (38.8%)	116 (51.8%)	21 (9.4%)		0.94 (0.63–1.39)
Sometimes	21	40.62 ± 6.04		8 (38.1%)	12 (57.1%)	1 (4.8%)		0.97 (0.38–2.45)
Often	4	31.00 ± 8.83		7 (53.8%)	6 (46.2%)	0 (0.0%)		0.51 (0.17–1.58)
Fixed-time feeding								
Never	78	40.21 ± 7.83	0.573	33 (42.3%)	42 (53.8%)	3 (3.8%)	0.649	1
Rarely	86	41.87 ± 8.03		30 (34.9%)	48 (55.8%)	8 (9.3%)		1.37 (0.73 ~ 2.57)
Sometimes	97	41.62 ± 8.72		34 (35.1%)	54 (55.7%)	9 (9.3%)		1.36 (0.74 ~ 2.51)
Often	130	40.42 ± 7.90		79 (40.5%)	97 (49.7%)	19 (9.7%)		1.08 (0.63 ~ 1.83)

*FAS* Feeding anxiety score

^a^ ORs value was calculated as combination of mild and severe anxiety (FAS > 38) compared with negative (FA ≤ 38) group

* *P* < 0.05

### Relations of maternal feeding anxiety with infant food refusal behaviors

The higher FAS was associated with infant food refusal behaviors, the maternal scores whose infant “never”, “rarely”, “sometimes” and “often” spat out food when feeding were 39.86 ± 8.02, 41.47 ± 8.18, 41.36 ± 7.44 and 42.14 ± 12.03 increasingly (*P* > 0.05), and the FA prevalence was significantly different among groups (*P* < 0.05), as shown in Table 5. The infants whose mother was identified as feeding anxiety were more likely to refuse opening the mouth when feeding (*P* < 0.05).

**Table 5:** Difference in maternal FAS and FA prevalence in relation to infant food refusal behaviors

Infant food refusal behaviors when feeding	N	FAS	*P* value	Feeding anxiety n(%)			*P* value	ORs(95% CI)^a^
		(mean ± SD)		Negative(≤38)	Mild(39 ~ 52)	Severe(≥53)		
Spitting out food								
Never	126	39.86 ± 8.02	0.436	54 (42.9%)	66 (52.4%)	6 (4.8%)	0.001^*^	1
Rarely	150	41.47 ± 8.18		58 (38.7%)	73 (48.7%)	19 (12.7%)		1.19 (0.73 ~ 1.93)
Sometimes	148	41.36 ± 7.44		47 (31.8%)	93 (78.2%)	8 (5.4%)		1.61 (0.98 ~ 2.64)
Often	28	42.14 ± 12.03		17 (53.1%)	9 (28.1%)	6 (18.8%)		0.66 (0.30 ~ 1.44)
Refusing to open mouth								
Never	250	40.51 ± 7.77	0.244	93 (37.2%)	144 (57.6%)	13 (5.2%)	0.026*	1
Rarely	147	41.23 ± 8.81		64 (43.5%)	63 (42.9%)	20 (13.6%)		0.77 (0.51 ~ 1.61)
Sometimes	54	42.93 ± 8.34		17 (31.5%)	31 (28.5%)	6 (11.1%)		1.29 (0.69 ~ 2.42)
Often	5	39.40 ± 8.08		2 (40.0%)	3 (60.0%)	0 (0.0%)		0.89 (0.15 ~ 5.42)
Turning heads aside								
Never	205	41.17 ± 7.87	0.809	70 (34.1%)	120 (58.5%)	15 (7.3%)	0.354	1
Rarely	155	40.63 ± 8.52		67 (43.2%)	71 (45.8%)	17 (11.0%)		0.68 (0.44 ~ 1.05)
Sometimes	89	41.48 ± 8.50		36 (40.4%)	46 (51.7%)	7 (7.9%)		0.76 (0.46 ~ 1.27)
Often	7	39.43 ± 7.70		3 (42.9%)	4 (51.7%)	0 (0.0%)		0.69 (0.15 ~ 3.18)
Crying								
Never	201	40.94 ± 7.90	0.955	75 (37.3%)	111 (55.2%)	15 (7.5%)	0.746	1
Rarely	168	40.90 ± 8.59		68 (40.5%)	83 (49.4%)	17 (10.1%)		0.88 (0.57 ~ 1.33)
Sometimes	82	41.39 ± 7.90		31 (37.8%)	45 (54.9%)	6 (7.3%)		0.98 (0.58 ~ 1.66)
Often	5	42.20 ± 13.41		2 (40.0%)	2 (40.0%)	1 (20.0%)		0.89 (0.15 ~ 5.47)

*FAS* Feeding anxiety score

^a^ ORs value was calculated as combination of mild and severe anxiety (FAS > 38) compared with negative (FA ≤ 38) group

* *P* < 0.05

### Multivariate analysis \

Multivariate linear regression was conducted to analyze the relations of maternal feeding anxiety with infant feeding behaviors. Variables included in the final model were determined by backward deletion method step by step with *P* < 0.05. The likelihood ratio F test was used for overall test of the model with result of F = 5.011, *P* < 0.001. After adjusting for maternal age, education level, household income, child-bearing number, gestational age and delivery mode, maternal FAS was positively related to infant bottle feeding and outdoor sunshine exposure practice (*P* < 0.05), and negatively related to household income level, as shown in Table 6.

**Table 6:** Multivariate analysis of maternal feeding anxiety as dependent variable

Independent variable	βi	*T*	*P* value	95% CI	
				Lower limit	Upper limit
Constant	42.820	10.139	< 0.001	34.520	51.119
Household income level	−1.083	−2.097	0.037	−2.098	−0.068
Colostrum feeding	−3.656	−1.934	0.054	−7.370	0.058
Bottle feeding	2.327	3.022	0.003	0.814	3.840
Outdoor sunshine exposure	1.824	2.410	0.016	0.337	3.311
Refusing to open mouth	0.846	1.655	0.099	−0.158	1.849

F = 5.011, *P* < 0.001

## Discussion

Our study indicated the maternal feeding anxiety was prevalent in China and 61.4% of women at 0–3 months postpartum had feeding anxiety symptoms, with 8.6% being severe anxiety. Maternal postpartum feeding anxiety was associated with negative infant feeding practices, like bottle feeding and infant food refusal behaviors. The results showed maternal mental health may be an important factor affecting child nutrition and health. Negative emotions during feeding may affect the maternal cerebral cortex, hypothalamus, and pituitary function, decrease secretion of prolactin and oxytocin, eventually reduce the breast milk secretion [6, 9].

Pregnancy and therefore lactation are important life events for women, during which their physiological and psychological state, as well as social status undergo considerable changes. The immediate change in life roles and new responsibilities after childbirth may be pressure provoking. Therefore, postpartum women are susceptible to anxiety, fear, and even depression [10]. Perinatal anxiety and depression are most common mental health problems in women. A systematic review summarized the prevalence and determinants of non-psychotic common perinatal mental disorders (CPMDs) in World Bank categorized low- and lower-middle-income countries, showed weighted mean prevalence was 15.6% (95% CI: 15.4–15.9) antenatally and 19.8% (19.5–20.0) postnatally, and the pooled prevalence of perinatal depression ranged from 5.2 to 32.9% during pregnancy and 4.9–59.4% after child birth [1]. In China the prevalence of postnatal anxiety or depression varies from 2.2 to 43.6% [11–14]. A systematic review of postpartum depression prevalence published during 1996–2012 showed the pooled prevalence was 14.7% (13.1–16.3%), increasing from the east of China to the west [15].

Given postpartum anxiety has been noted as having independent effects just as postpartum depression, and significant comorbidity has also been noted between postpartum anxiety and depression, it is surprising that so little research has been conducted on postpartum anxiety, compared with postpartum depression. A narrative review showed [5] the prevalence of postpartum anxiety (ranged from 13 to 40%) has varied according to the definition, the anxiety scale used, the cut-off scores on the scales, the severity of anxiety, the timing of the assessment, the recruitment sample and the origin (country) of research. In this study, the Self-rating Feeding Anxiety Scale (SFAS) was used to assess the maternal postpartum anxiety, which was developed specifically to tap postnatal-specific anxiety-feeding anxiety, and reliability and validity has been tested [13]. Besides assessment tools, the cultural and socio-economic settings, sample sizes, cutoff point used, and timing of assessment affect prevalence. In this study the maternal feeding anxiety was assessed at 0 ~ 3 months postpartum, which was during the Chinese traditional “doing the month” period, both limited socialization and family support could worsen maternal mental problems and lead to higher anxiety symptom prevalence. In fact, the severe FA prevalence was only 8.6% (FAS>52), that was similar to other studies. In order to achieve reliable results globally, there is a need to establish widely accepted assessment tools, cutoff scores, and timing of assessment.

Systematic review showed the correlates factors for postpartum anxiety included demographic factors, childbirth experiences, social support and history of psychiatric and psychological problems [1]. The demographic risk factors for postnatal anxiety include being a young mother, having more education and being employed. Childbirth risk factors include being primiparous in one sample and multiparous in another, caesarean delivery, fear of the birth and of death during delivery, lack of control during labor, low self-confidence for the delivery and the delivery staff, and premature delivery. Social support problems include the lack of family support, marital/family conflict, and social health issues. Psychiatric history risk factors include prenatal depression and anxiety. Several studies have concluded that preterm birth and cesarean section are independent risk factors of postpartum anxiety [16–18] We found a higher FAS in women with younger age, lower household income, first-time mother and preterm birth, but without statistical significance (*P* > 0.05*)*, and FA prevalence was associated with delivery mode (*P* < 0.05*)*. Trumello et al. [19] found that mother-child separation caused by the need for specialized care of premature infants may exert a negative effect on the mothers’ emotional state; thus, women who give birth to premature infants are more likely to develop postpartum anxiety. A Chinese study [20] found that postpartum women aged ≥35 were more prone to anxiety or depression. Ye et al. (2014) [21] reported a higher prevalence of postpartum anxiety among mothers with higher education levels. Zhang et al. [11] found that women were more vulnerable to feeding anxiety with lower household income. Bina R et al. (2017) [22] found that postpartum women with lower incomes reported more symptoms of anxiety. And another study showed that postpartum women’s perceived difficulty managing on household income was associated with anxiety symptomatology [23]. Other studies have suggested that social psychological factors, such as emotional support from family and society, and various expectations of the sex of the newborn also considerably affected the mother’s feelings during feeding [11, 24]. Because of the limited data we haven’t analyzed the relations of FA with family support and history of psychiatric and psychological problems, that is the study limitation.

Postpartum anxiety are always associated with unpleasant emotional experiences, such as worry, fear, irritability, and frustration, that can last for a few weeks or even longer [25]. Maternal anxiety during feeding may eradicate any willingness to breastfed and even affect the composition of the breast milk, ultimately affecting the growth and development of infant. The systematic literature review included negative effects on breast-feeding, bonding, mother–infant interactions, infant temperament, sleep, mental development, health and internalizing in infants and on conduct disorder in adolescents, based on structured clinical interviews and behavior observations [1].

Our study indicated higher FA risk seemed to be found in women who did not feed infant colostrum or fed infant with bottle. This may be explained by that mothers who fed infant colostrum could generally let baby touch breast in mother’s arms within 1 h after delivery, which promoted emotional communication between mother and infant and helped breastfeeding. However bottle feeding might reduce mother-child contact, and hinder emotional communication during feeding. Furthermore, bottle feeding led to insufficient breast milk secretion and subsequent interruption of breastfeeding, which diminished maternal confidence in infant feeding. On the other hand, bottle feeding might help mothers control the milk intake of infants, and mothers with FA may prefer bottle feeding to breastfeeding. Lower FA prevalence was found in women who breastfed exclusively but without statistical significance. These findings are supported by two studies, in which mothers who breastfed are less likely to develop FA, as compared to mothers who chose formula feeding, and mothers suffered from FA does not emerge favorable persistence signs associated with breastfeeding [26, 27].

Moreover, mothers also had a significantly higher FAS if their babies had been exposed to sunshine outdoors. A possible explanation may be that mothers with FA might be more cautious about the infant’s health so they spent more time with their infants outdoors to synthesize vitamin D. Infant food refusal behaviors may reduce maternal confidence, and induce mother’s anxiety about infants’ nutrient intake and development. But above results needed to be confirmed by more research in the future, because the limitations of sample size and sampling method decreased the power of test.

Despite our results and the mounting evidence indicating high prevalence of maternal mental health and its adverse impact on infant feeding practices, the maternal mental health problems has not been focused. Consequently the treatment gap for mental illness is large, accounting for 76–85% patients with mental health not receiving intervention. Studies show that psychosocial, educational, and supportive interventions are effective in improving maternal mental health. That will be our next research direction, to explore the effective intervention strategy to improve maternal mental health and early child development. And theoretically the anesthesia type during delivery would have an influence on breastfeeding, but regrettably the related information has not been collected in this study. In the future, if possible we will investigate the data retrospectively and compensatively in the other relevant investigation.

Several limitations in our study deserved acknowledgements, like convenient sampling, small sample size, unpopular assessment tool, related factors (family support) data deficiency, etc.

## Conclusion

Maternal FA prevalence were 61.4% (FAS>38) with severe FA being 8.6% (FAS>52) at 0–3 months postpartum. Maternal postpartum feeding anxiety was associated with some infant feeding practices, including bottle feeding and infant food refusal behaviors.

## Supplementary Information

**Additional file 1.** Questionnaire of assessment of feeding anxiety.

**Additional file 2.** Questionnaire of infant feeding practices (FPs) investigation.

**Additional file 3.** Questionnaire of demographic characteristics.

## Data Availability

The datasets used and/or analyzed during the current study are available from the corresponding author on reasonable request.

## Abbreviations

FA: Feeding Anxiety
FPs: Feeding Practices
FAS: Feeding Anxiety Scores
SFAS: Self-rating Feeding Anxiety Scale
ORs: Odds Ratios
CI: Confidence Intervals
