# Supplementary material for: Maternal postpartum feeding anxiety was associated with infant feeding practices: results from the mother-infant cohort study of China
Source: BMC Pregnancy Childbirth. 2020 Dec 14;20:780. doi: 10.1186/s12884-020-03483-w (PMC7737271; doi:10.1186/s12884-020-03483-w)
Supplement: Supplementary file 1 — Additional file 1. Questionnaire of assessment of feeding anxiety. [file 12884_2020_3483_MOESM1_ESM.docx]

**Supplementary file**

Supplementary file 1: Questionnaire of assessment of feeding anxiety

Please read each of 23 sentences below which describe mothers’ emotion during feeding. Basing on your real fact (emotion) in the past week, please choose appropriate answer and write “√” in the right blank. The answers of the questions are not belong to right answer and wrong answer, the only request for you is that finishing the questionnaire basing on your real fact. You can finish the questionnaire as soon as possible, but avoid forgetting writing some part of the questionnaire. We genuinely appreciate for your cooperation.

| Number | Question | ①never | ②sometimes | ③often | ④all time |
| --- | --- | --- | --- | --- | --- |
| 1 | I was annoyed by infant’s crying. | ① | ② | ③ | ④ |
| 2 | I worried about infant picky eating. | ① | ② | ③ | ④ |
| 3 | I concerned about the cost of feeding infant. | ① | ② | ③ | ④ |
| 4 | I worried about infant eating not enough. | ① | ② | ③ | ④ |
| 5 | I was tired due to problem of feeding infant. | ① | ② | ③ | ④ |
| 6 | I couldn’t sleep during night because of thinking problem of feeding infant frequently. | ① | ② | ③ | ④ |
| 7 | Feeding infant made me communicate well with infant. | ① | ② | ③ | ④ |
| 8 | I really hoped that feeding infant wasn’t so annoying. | ① | ② | ③ | ④ |
| 9 | I cared about other mothers’ thinking of my feeding pattern. | ① | ② | ③ | ④ |
| 10 | I worried that improper feeding would influence infant’s health. | ① | ② | ③ | ④ |
| 11 | If I thought family members or nanny used incorrect feeding pattern, I was worried. | ① | ② | ③ | ④ |
| 12 | I was stressful when I communicated feeding experience with other mothers. | ① | ② | ③ | ④ |
| 13 | I worried that infant’s tableware and milk bottle were unclean. | ① | ② | ③ | ④ |
| 14 | I made a decision difficultly when I encountered problem of feeding infant. | ① | ② | ③ | ④ |
| 15 | I hesitated to choose food for infant. | ① | ② | ③ | ④ |
| 16 | I thought I was able to be a mother successfully. | ① | ② | ③ | ④ |
| 17 | I could do as well as other mothers in the problem of feeding infant. | ① | ② | ③ | ④ |
| 18 | Taking good care of infant was a big challenge for me. | ① | ② | ③ | ④ |
| 19 | I worried that improper feeding impacted infant’s intellectual development. | ① | ② | ③ | ④ |
| 20 | I was stressed about infant food safety. | ① | ② | ③ | ④ |
| 21 | I couldn’t eat and sleep well when infant ate not well. | ① | ② | ③ | ④ |
| 22 | I was worried when infant ate food given by others. | ① | ② | ③ | ④ |
| 23 | I thought it was all my fault when infant had poor appetite. | ① | ② | ③ | ④ |
