# Supplementary material for: Maternal postpartum feeding anxiety was associated with infant feeding practices: results from the mother-infant cohort study of China
Source: BMC Pregnancy Childbirth. 2020 Dec 14;20:780. doi: 10.1186/s12884-020-03483-w (PMC7737271; doi:10.1186/s12884-020-03483-w)
Supplement: Supplementary file 2 — Additional file 2. Questionnaire of infant feeding practices (FPs) investigation. [file 12884_2020_3483_MOESM2_ESM.docx]

Supplementary file 2: Questionnaire of infant feeding practices (FPs) investigation

1. How long did infant move to mother’s arm and touch mother’s breast? (sucking and lactating was unnecessary.)

①<1 hour ②1—23 hours ③≥24 hours ⑨ Forgetting/Unknowing

1. Did you give infant colostrum? (Colostrum which has thick and yellow appearance is milk lactating in 7 days after delivery)

①No ②Yes

1. During the first 3 days of infant, did you feed other food or liquid before breastfeeding?

①No ②Yes

4. From birth to age of 6 months age of infant, which feeding method was used?

①Exclusive breastfeeding (Feeding no other food and liquid, excluding breast milk, vitamins, rehydration salts)

②Mixed feeding (giving breast milk along with other dairy, semi-solid or solid food)

③Artificial feeding (not giving breast milk)

5. Did you feeding infant water, drink of juice yesterday (including day and night)?

①No ②Yes

6. Infant drank ______ time non-breast milk (including formula/non-formula/milk/goat's milk/yogurt, etc.) yesterday (including day and night).

7. Did infant eating food (including breast milk, water, other milk or supplementary food, etc.) by a bottle with pacifier yesterday (including day and night)?

①No ②Yes

8. During the past month, which was the frequency of occurring following feeding behavior between feeding person and infant? (Please write “√” in the right blank)

| Feeding behavior | Frequency | | | | |
| --- | --- | --- | --- | --- | --- |
| 1. Feeding infant as more as possible | 1.Never | 2.Rarely | 3.Sometimes | 4.Often | 5.Always |
| 2. Perceiving infant hungry and satiety signs |  |  |  |  |  |
| 3. Feeding infant at fixed-time |  |  |  |  |  |
| 4. Infant spitting out food |  |  |  |  |  |
| 5. Infant refusing to open mouth |  |  |  |  |  |
| 6. Infant turning heads aside to avoid eating |  |  |  |  |  |
| 7. Infant crying and avoid eating |  |  |  |  |  |

9. During outdoor activities, did infant expose his or her skin to the sunshine?

①No ②Yes
