# Supplementary material for: Maternal postpartum feeding anxiety was associated with infant feeding practices: results from the mother-infant cohort study of China
Source: BMC Pregnancy Childbirth. 2020 Dec 14;20:780. doi: 10.1186/s12884-020-03483-w (PMC7737271; doi:10.1186/s12884-020-03483-w)
Supplement: Supplementary file 3 — Additional file 3. Questionnaire of demographic characteristics. [file 12884_2020_3483_MOESM3_ESM.docx]

Supplementary file 3: Questionnaire of demographic characteristics

1. Your date of birth (year/month/day): ______ Note: Please write the Gregorian calendar.

2. What is your level of education?

①Never went to school

②Primary school

③Junior high school

④High school/Technical secondary school

⑤College

⑥University

⑦Graduate students and above

3. In the past year, what was monthly income per capita of your family? (including items and rental income, etc.)

①Less than RMB 500

②RMB 501-1000

③RMB 1001-2000

④RMB 2001-3000

⑤RMB 3001-4000

⑥RMB 4001-6000

⑦RMB 6001-8000

⑧More than RMB 8000

⑨Unknown

4. This was your ______ time delivery.

5. Your infant was born at ______ weeks.

6. Which delivery mode was used in this delivery?

①Vaginal delivery

②Assisted delivery (side incision, forceps)

③Cesarean
